# Autism spectrum-related symptoms and clinical ASD diagnoses in children and adolescents with classical galactosemia: a descriptive clinical cohort study

**DOI:** 10.3389/fpsyt.2026.1836428

**Published:** 2026-07-21

**Authors:** Aleksandra Gozdanek, Amanda Krzywdzińska-Rogowska, Barbara Remberk, Anna Bauer, Jolanta Sykut-Cegielska

**Affiliations:** 1 Department of Child and Adolescent Psychiatry, Institute of Psychiatry and Neurology, Warsaw, Poland; 2 Department of Inborn Errors of Metabolism and Pediatrics, Institute of Mother and Child, Warsaw, Poland

**Keywords:** autism spectrum disorder, classical galactosemia, inborn errors of metabolism, long-term outcomes, neurodevelopmental disorders, psychiatric manifestations

## Abstract

**Background:**

Classical galactosemia (CG) is a rare inherited metabolic disorder associated with long-term neurodevelopmental, language, cognitive and psychosocial difficulties. Social-communication problems may overlap clinically with autism spectrum disorder (ASD), but paediatric data based on standardised ASD assessment remain limited.

**Methods:**

The study included 50 children and adolescents aged 6–17 years with confirmed classical galactosemia. Intellectual functioning was assessed using the Stanford–Binet Intelligence Scales, Fifth Edition. Autism spectrum disorder (ASD) symptoms were evaluated using the Mini International Neuropsychiatric Interview for Children and Adolescents (MINI-KID), Autism Spectrum Rating Scales (ASRS, parent version), Autism Diagnostic Observation Schedule, Second Edition (ADOS-2), as well as comprehensive clinical assessment by a child and adolescent psychiatrist.

**Results:**

A clinical diagnosis of ASD was established in 36% of participants. Results indicating ASD symptoms were obtained in 56% of children using MINI-KID, 48% using ADOS-2, and 30% based on overall ASRS scores. Statistically significant moderate negative correlations were found between IQ and ASD symptom severity, particularly in social communication, social-emotional reciprocity, and attention domains. Selected long-term disease-related manifestations (white matter abnormalities and osteopenia/osteoporosis) were more frequent in children with ASD symptoms identified by MINI-KID.

**Conclusions:**

Children and adolescents with classical galactosemia in this clinical cohort frequently presented ASD-related social-communication difficulties, and a substantial proportion met criteria for a clinical ASD diagnosis.

## 1 Introduction

Autism spectrum disorder (ASD) is a neurodevelopmental condition characterised by persistent difficulties in social communication and interaction, accompanied by restricted and repetitive patterns of behaviour, interests or activities (1). The clinical presentation of ASD is highly heterogeneous and may coexist with intellectual, language and other neurodevelopmental difficulties (2, 3).

According to the most recent estimates, ASD affects approximately 1–2% of the general population worldwide (3, 7). Reported prevalence rates vary across studies, partly due to differences in diagnostic practices, case ascertainment and methodological approaches (4).

Symptoms of ASD most often become apparent in early childhood, but diagnosis may be delayed, especially in high-functioning individuals, whose symptoms only become more apparent in highly demanding social situations (2). An additional diagnostic difficulty is the frequent co-occurrence of ASD with other neurodevelopmental and psychiatric disorders, such as ADHD, anxiety disorders, intellectual disability or speech disorders (2). Among the causes of developmental delays are metabolic diseases and rare genetic syndromes that modify the expression of autistic symptoms or hinder their diagnosis (5, 6).

Classical galactosemia (CG) is a rare metabolic disease inherited in an autosomal recessive manner, belonging to the group of inborn defects of carbohydrate metabolism. Its cause is a deficiency of the enzyme galactose-1-phosphate uridyltransferase (*GALT*), leading to an accumulation of galactose and its metabolites (5). The average prevalence of CG in Europe is estimated to be 1:23 000-1:44–000 births, whilst in Poland it is approximately 1:35–000 births (8, 9).

The first symptoms of CG appear in newborns after ingestion of lactose-containing milk and include hepatopathy, hypoglycaemia, hypotonia, sepsis and cataracts (5, 10). Despite an early introduction of a galactose elimination diet, most patients develop chronic complications such as speech developmental delay, cognitive impairment, tremors, demyelinating changes, osteopenia or primary ovarian failure in girls (11, 12). Importantly, these complications develop independently of adherence to dietary treatment, indicating the complex pathophysiology of the disease and its long-term consequences (6).

Beyond somatic complications, classical galactosemia is associated with a broad range of neurodevelopmental and cognitive difficulties. Data from the GalNet registry indicate that speech and language disorders, cognitive impairment and neurological complications are amongst the most common long-term outcomes of the disease (11). These difficulties may significantly affect everyday communication, social functioning and adaptive behaviour.

An important clinical challenge is that several neurodevelopmental features frequently observed in CG, particularly language impairment, social communication difficulties and cognitive dysfunction, may resemble core ASD symptoms. Consequently, distinguishing between true ASD and ASD-like manifestations associated with the neurodevelopmental phenotype of CG may be difficult in routine clinical practice (11, 24).

Previous studies in classical galactosemia have consistently reported impairments in social functioning and broader cognitive functioning that may contribute to difficulties in interpersonal communication (11, 24). However, it remains unclear to what extent these manifestations represent autism spectrum disorder itself or reflect the broader neurodevelopmental phenotype associated with CG.

Despite the growing interest in the relationship between CG and ASD, studies to date have predominantly focused on adult patient populations and have been descriptive in nature (12–16).

Data regarding ASD in children and adolescents with CG remain limited. In particular, few studies have systematically evaluated ASD-related symptoms using standardised assessment tools whilst simultaneously considering the potential overlap between ASD and the characteristic cognitive, language and social communication difficulties associated with CG (11, 24).

The primary aim of this study was to describe the frequency and clinical profile of ASD-related symptoms and final clinical ASD diagnoses in a cohort of children and adolescents with classical galactosemia. Secondary aims were: (1) to compare the proportion of children identified as having ASD-related symptoms across different assessment sources (ASRS, ADOS-2, MINI-KID and final psychiatric diagnosis); (2) to examine associations between intellectual functioning and severity of ASD-related symptoms; and (3) to explore whether selected long-term complications of CG are associated with ASD-related symptoms or final clinical ASD diagnosis.

## 2 Materials and methods

### 2.1 Eligibility criteria and research procedure

50 patients with a diagnosis of classical galactosemia (CG) under the care of the Department of Inborn Errors of Metabolism and Pediatrics of the Institute of Mother and Child in Warsaw, which looks after the vast majority of CG patients in Poland, were enrolled in the study. Despite the lack of a national registry dedicated to the disease, available clinical data indicate that the facility provides care for approximately 90% of all CG patients.

Inclusion criteria:

- confirmed diagnosis of classical galactosemia, based on:
- significantly reduced or undetectable activity of the enzyme galactose-1-phosphate uridyltransferase (*GALT*) in erythrocytes, *and/or*
- presence of two pathogenic *GALT* gene variants, *and/or*
- characteristic clinical picture in the neonatal period combined with an abnormal metabolic profile (elevated galactose-1-phosphate or galactose),
- child age 6–17 years,
- consent of parent/guardian and consent of patient over 13 years of age to participate in the study.

Exclusion criteria:

- lack of confirmed diagnosis of classical galactosemia;
- age outside the 6–17 years range;
- lack of informed consent from the legal guardian or, where applicable, lack of assent from the participant.

During participant recruitment, no factors were identified that could compromise the completion or reliable interpretation of the diagnostic procedures used in the study.

The study was conducted between 2022 and 2024 at the Department of Inborn Errors of Metabolism and Pediatrics of the Mother and Child Institute in Warsaw (internal grant of the Mother and Child Institute in Warsaw, OPK 510-06-05). The project was approved by the Bioethics Committee of the Mother and Child Institute. Written informed consent was obtained from all participants. In the case of minors, consent was signed by legal guardians; in addition, in patients over 13 years of age, their own consent was also obtained (so-called double consent).

Due to the rare prevalence of CG (estimated at 1:23 000-1:44–000 births in Europe, and approximately 1:35–000 in Poland) (5, 8, 9) and the fact that a large proportion of known paediatric cases of the disease in Poland were included in the study, as well as the organisational constraints and ethical difficulties associated with conducting in-depth psychiatric diagnosis in healthy children, a control group was not included in this study. This approach is justified in the literature on rare disease research, where, due to limited sample sizes and high value of each individual observation, descriptive studies, comparisons against population data or data from international registries such as GalNet (10, 11, 13) are often used.

Therefore, all findings are presented as descriptive frequencies within this clinical cohort and should not be interpreted as population prevalence estimates.

### 2.2 Diagnostic tools

The following tools were used to assess autistic symptoms, intellectual functioning and co-occurring psychiatric symptoms:

- Stanford-Binet Scale, Fifth Edition (SB5) – an individual, widely used test assessing general intelligence and cognitive ability in children and adolescents (18). The study used the full version of the SB5, covering five domains: fluid reasoning, knowledge, quantitative reasoning, visuospatial processing and working memory, analysed in verbal and non-verbal components. The scale has high validity and reliability. Its Polish adaptation is based on standardised normative data.
- Autism Spectrum Rating Scales (ASRS) – a standardised screening questionnaire for the assessment of ASD symptoms in children and adolescents aged 2–18 years, developed on the basis of the DSM-IV-TR criteria (19). Assesses a wide range of behaviours associated with ASD including deficits in communication skills, difficulties with peer and adult relationships, behavioural rigidity, sensory sensitivity, self-regulation, attention deficits, stereotypic behaviours. Self-report, parent and teacher versions are available; the parent version of the Polish adaptation of the tool was used in this study.
- Autism Diagnostic Observation Schedule, Second Edition (ADOS-2) – a standardised, semi-structured observation tool considered to be the gold standard for assessing ASD symptoms (20). It consists of five modules adapted to age and level of language development. The study used modules appropriate to each participant, in accordance with the guidelines of the manual. The assessment was carried out by certified diagnosticians on the basis of direct observation. Results were analysed using diagnostic algorithms and comparison scales to classify and assess the severity of ASD symptoms.
- Mini International Neuropsychiatric Interview for Children and Adolescents (MINI-KID), version 7.0.2 – a structured diagnostic interview based on ICD-10 and DSM-5 criteria to assess mental disorders in children and adolescents aged 6–17 years (21). As part of the research project, the MINI-KID was conducted with all participants to assess their mental functioning holistically. The analysis focused exclusively on data relating to symptoms on the autism spectrum. Although MINI-KID is not a dedicated tool for ASD diagnosis, its use was complementary and exploratory to other diagnostic methods such as ADOS-2 and ASRS.

All standardised test results were interpreted by a child and adolescent psychiatrist in the context of developmental history, parent interview, psychiatric examination, cognitive assessment and medical records. The final clinical ASD diagnosis was based on the overall clinical picture and current ICD-10 and DSM-5 diagnostic criteria, not on any single instrument. Particular caution was applied when interpreting behaviours that could be influenced by CG-related language impairment, speech disorders, sensory symptoms, motor abnormalities or intellectual functioning.

In addition, information obtained from the medical records of patients of the Department of Inborn Errors of Metabolism and Pediatrics of the Mother and Child Institute in Warsaw was included in the study. Data included demographics, genetic test results and distant complications of the disease, i.e.: neurological disorders, speech disorders, primary ovarian failure, cataracts, bone mineralisation disorders. Information regarding speech and language disorders was obtained from neurological speech-language assessments documented in the patients’ medical records. Data regarding demyelination or hypomyelination were based on neuroimaging findings documented in the medical records.

### 2.3 Statistical analysis

Statistical analysis was carried out using IBM SPSS Statistics 29. At the initial stage, the completeness of the data was verified and the distributions of the variables were assessed. All analysed data came from complete cases – all participants underwent the planned diagnostic procedures, which allowed for a fully comprehensive analysis without the need for data imputation.

The analytical procedures used included:

- Spearman’s rank correlation test – to assess associations between intellectual functioning (IQ) and ASD-related symptom severity measured using ASRS scores,
- Mann-Whitney U-test – for comparisons of quantitative outcomes (e.g. IQ, screening scale scores) between groups of children with and without an ASD diagnosis,
- chi-square test – to analyse the relationship between qualitative variables such as gender, the presence of complications (e.g. osteopenia, demyelinating lesions) and the presence of ASD symptoms,
- ASD-related symptom frequency analysis - separately for each assessment source (ASRS, ADOS-2, MINI-KID and final psychiatric diagnosis).

In the case of significant differences, measures of the strength of the effect (e.g. Phi or r coefficient) were also calculated. The level of statistical significance was set at p<0.05. All tests were two-tailed.

### 2.4 Characteristics of the study group

All patients underwent an enzymatic assay, which showed galactose-1-phosphate uridyltransferase (*GALT*) activity in the range of 0-1% of the mean population activity. In parallel, molecular analysis was performed, identifying pathogenic variants in the *GALT* gene. 68% of patients had the p.Gln188Arg or p.Lys284Asn variant in either a homozygous or heterozygous pattern. In the remaining 32%, other less common pathogenic variants of the *GALT* gene were detected (9).

The demographic characteristics of the study sample are shown in **Table 1**. The majority of participants came from rural areas or towns with up to 50,000 inhabitants. The mean age of the children was 12.02 years (SD = 4.07). The mean age of mothers at birth was 29.02 years (SD = 4.48) and of fathers was 31.66 years (SD = 5.79).

**Table 1:** Characteristics of study sample (N = 50).

Variables	*N*	%
Place of residence		
Rural	20	40.0
City up to 50,000	18	36.0
City up to 100,000	5	10.0
City between 101,000 and 250,000	3	6.0
City between 251,000 and 500,000	1	2.0
City over 500,000	3	6.0
Mother’s education		
Primary	0	0
Lower-secondary	4	8.0
Basic vocational	9	18.0
Upper-secondary	13	26.0
Higher education	24	48.0
Father’s education		
Primary	1	2.0
Lower-secondary	2	4.0
Basic vocational	13	26.0
Upper-secondary	13	26.0
Higher education	21	42.0
Sex of child		
Boy	26	52.0
Girl	24	48.0
	*M*	*SD*
Age of child	12.02	4.07
Mother’s age	29.02	4.48
Father’s age	31.66	5.79

There were comparable numbers of boys (52%) and girls (48%) in the study group. Higher education qualifications were declared by 48% of mothers and 42% of fathers.

Most of the children were of school age, which is reflected in the average age of 12 years. The relatively young age of the parents is also noted. Most families lived in rural areas and small towns. The gender proportions of the study group were similar.

The demographic and genetic profiles of the study group are similar to data reported in paediatric studies on galactosemia (22) and consistent with characteristics presented in analyses of international patient registries (11, 13).

## 3 Results

### 3.1 Level of intellectual functioning

The children and adolescents’ level of intellectual functioning was assessed using the Stanford-Binet Scale, fifth edition (SB5). The results are shown in **Table 2**. The largest group were patients with an average level of intelligence (44%). Next were those with below-average intelligence (16%) and with borderline scores in the IQ range of 70-84 (14%).

**Table 2:** Characteristics of intelligence level of participating children – frequency analysis.

Level of intelligence	*N*	%
Moderate intellectual disability	1	2.0
Mild intellectual disability	7	14.0
Boundary score between norm and disability	7	14.0
Below average intelligence	8	16.0
Average intelligence	22	44.0
Above-average intelligence	5	10.0

Intellectual disability was found in 8 participants (16%). Of these, 7 children (14%) met the criteria for mild intellectual disability, whilst 1 child (2%) scored equivalent to moderate disability. This indicates that the majority of patients presented a normal or close to normal range of cognitive functioning.

### 3.2 Symptoms on autism spectrum

Four independent data sources were used to assess autistic symptoms: the ASRS questionnaire, the ADOS-2 observational scale, the MINI-KID structured interview used as an exploratory complementary source and the clinical diagnosis done by a children’s psychiatrist.

The results of the ASRS questionnaire indicate that a significant proportion of children showed elevated or high levels of symptoms in various domains. In the area of “Social Relationships and Communication”, a total of 30% of children scored in the elevated or higher range, and 30% in the area of “Atypical Behaviours”. In the “Self-regulation” category, 38% of respondents scored above average, whilst in “Overall performance” – it was 30%. On the DSM Scale, values in the elevated and higher range were reached by 34% of respondents.

In the detailed analysis of the subscales: 50% of children showed elevated or high scores in the area of “Relationships with peers”, 38% in “Relationships with adults” and 32% in “Social and emotional reciprocity”. In terms of “Atypical language”, 30% of the children had elevated or higher scores. Symptoms related to movement stereotypies were less common, with only 4% of children scoring in the very high range. In the subscale of “Rigidity in behaviour”, 20% of respondents scored high or very high, and in “Sensory sensitivity” – it was 28%. In the area of “Attention”, 40% of children obtained scores indicating increased difficulties (**Table 3**).

**Table 3:** Response characteristics of the ASRS Scale – frequency analysis.

ASRS results	Low		Average		Elevated		High		Very high	
	*n*	*%*	*n*	*%*	*n*	*%*	*n*	*%*	*n*	*%*
Social relations / communication	9	18.0	26	52.0	6	12.0	6	12.0	3	6.0
Atypical behaviour	12	24.0	23	46.0	4	8.0	7	14.0	4	8.0
Self-regulation	0	0	31	62.0	10	20.0	6	12.0	3	6.0
Overall performance	5	10.0	30	60.0	4	8.0	4	8.0	7	14.0
DSM scale	15	30.0	18	36.0	5	10.0	3	6.0	9	18.0
Relationships with peers	3	6.0	22	44.0	6	12.0	6	12.0	13	26.0
Relationships with adults	2	4.0	29	58.0	7	14.0	7	14.0	5	10.0
Social and emotional reciprocity	12	24.0	22	44.0	2	4.0	9	18.0	5	10.0
Atypical language	3	6.0	32	64.0	5	10.0	7	14.0	3	6.0
Stereotypies	26	52.0	21	42.0	1	2.0	0	0	2	4.0
Rigidity in behaviour	16	32.0	21	42.0	3	6.0	5	10.0	5	10.0
Sensory sensitivity	2	4.0	24	48.0	10	20.0	7	14.0	7	14.0
Attention	1	2.0	29	58.0	9	18.0	6	12.0	5	10.0

When assessed with the ADOS-2 diagnostic algorithm, low severity of autistic symptoms was found in 12% of children, moderate in 24% and high in 12%. A total of 48% of subjects met the criteria for ASD (**Table 4**).

**Table 4:** Characteristics of ADOS-2 results – frequency analysis.

Diagnosis of ASD according to ADOS-2	Level of symptoms	*N*	%
No	None or minimal symptoms	26	52.0
Yes	Low	6	12.0
Yes	Moderate	12	24.0
Yes	High	6	12.0

In the structured MINI-KID interview, ASD-related symptoms were indicated in 56% of children. In terms of the clinical diagnosis made by a psychiatrist based on a holistic assessment of functioning and the results of all diagnostic tools, a diagnosis of ASD was made in 36% of the study participants.

**Figure 1:**
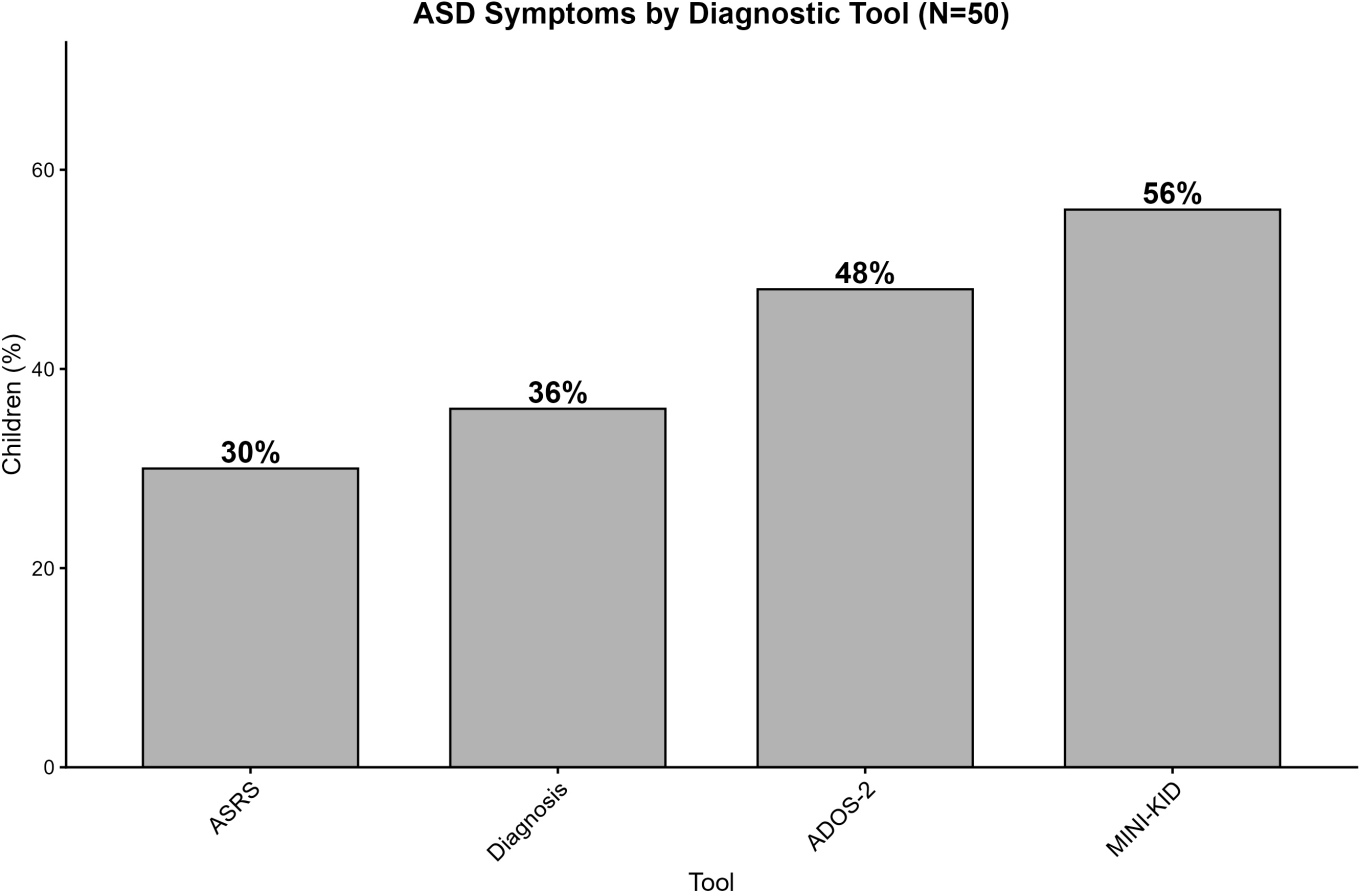
Frequency of autism spectrum disorder (ASD) related symptoms according to the diagnostic tool used (N = 50).

**Figure 1** summarises the frequency of ASD-related findings across the four assessment sources. The highest proportion was obtained in the exploratory MINI-KID interview, followed by ADOS-2 and final psychiatric diagnosis, whereas the ASRS overall score identified the lowest proportion of elevated results. These differences reflect the distinct purpose and format of each assessment source and are interpreted further in the Discussion.

### 3.3 Analysis of the relationship between intelligence level and severity of ASD symptoms

In order to investigate the relationship between intellectual functioning (IQ) and the severity of autistic symptoms, Spearman rank correlation analysis was performed for the ASRS questionnaire scores. The results showed statistically significant negative correlations between IQ level as measured by the SB5 and symptom severity across multiple ASRS subscales. The strongest correlations were in the areas of: “Social relationships and communication” (r_s_ = -0.42, p = 0.002), “Overall performance” (r_s_ = -0.34, p = 0.015), “DSM scale” (r_s_ = -0.37, p = 0.009), “Relationships with peers” (r_s_ = -0.32, p = 0.022), “Social and emotional reciprocity” (r_s_ = -0.38, p = 0.006) and “Attention” (r_s_ = -0.40, p = 0.004).

Similarly, significant correlations were also obtained when analysing IQ ranges - for example, for Relationships with adults (r_s_ = -0.33, p = 0.020) and Social and emotional reciprocity (r_s_ = -0.41, p = 0.004).

The results of the detailed analyses are shown in **Table 5**. Weak to moderate correlations were obtained for most dimensions, indicating that higher IQ levels were associated with lower severity of autistic symptoms.

**Table 5:** Spearman correlations between intelligence level and severity of autism symptoms.

	SB-V full scale		IQ range	
	*r_s_*	*p*	*r_s_*	*p*
social relations / communication	-0.42	0.002	-0.44	0.001
atypical behaviour	-0.19	0.198	-0.18	0.209
self-regulation	-0.19	0.181	-0.24	0.099
overall performance	-0.34	0.015	-0.36	0.010
DSM scale	-0.37	0.009	-0.38	0.007
relationships with peers	-0.32	0.022	-0.34	0.015
relationships with adults	-0.27	0.059	-0.33	0.020
social and emotional reciprocity	-0.38	0.006	-0.41	0.004
atypical language	-0.27	0.055	-0.22	0.119
stereotypies	-0.27	0.058	-0.27	0.056
rigidity in behaviour	-0.17	0.230	-0.16	0.263
sensory sensitivity	0.03	0.813	0.02	0.877
attention	-0.40	0.004	-0.42	0.003

In addition, an analysis of the data categorised by ASRS classification bands was carried out. The results presented in **Table 6** confirm the presence of negative correlations between intelligence level and symptom severity on most dimensions – most strongly on social relationships, emotional reciprocity, attention and the DSM scale. No significant associations were noted for atypical behaviour, atypical language, rigidity in behaviour and sensory sensitivity, whereas weak but statistically significant correlations were observed for stereotypies.

**Table 6:** Spearman correlations between intelligence level and severity of autism symptoms - categorised data.

	SB-V full scale		IQ range	
	*r_s_*	*p*	*r_s_*	*p*
Social relations/communication	-0.36	0.009	-0.40	0.004
Atypical behaviour	-0.20	0.163	-0.20	0.173
Self-regulation	-0.33	0.018	-0.34	0.015
Overall performance	-0.31	0.029	-0.35	0.014
DSM scale	-0.34	0.017	-0.36	0.009
Relationships with peers	-0.31	0.028	-0.34	0.014
Relationships with adults	-0.35	0.012	-0.39	0.005
Social and emotional reciprocity	-0.32	0.023	-0.37	0.009
Atypical language	-0.25	0.079	-0.23	0.108
Stereotypies	-0.28	0.047	-0.29	0.042
Rigidity in behaviour	-0.19	0.179	-0.19	0.185
Sensory sensitivity	-0.02	0.884	-0.07	0.628
Attention	-0.39	0.006	-0.33	0.018

The observed results indicate a significant relationship between the level of intellectual functioning and the severity of autistic symptoms, particularly in the areas of social competence, attention and emotional reciprocity.

### 3.4 Distant complications of galactosemia and their relationship to ASD symptoms

In the study group of children with classical galactosemia, a diverse range of distant complications was observed. The most common neurological complications were white matter lesions – demyelination or hypomyelination – present in more than half of patients (52%). Speech disorders were present in 50% of children and delayed speech development in 48%. Neurological symptoms in the form of intention or rest tremor were reported in 32% of the subjects. Endocrine and metabolic problems included ovarian failure in 28% of girls and osteopenia or osteoporosis in 28% of all children. Cataracts in neonatal period were diagnosed in 8% of patients and intellectual disability in 16% (**Table 7**).

**Table 7:** Characteristics of the incidence of distant complications of galactosemia – frequency analysis.

complications	*N*	%
Demyelination/hypomyelination	26	52.0
Speech disorders	25	50.0
Delayed speech development	24	48.0
Intentional/spontaneous tremors	16	32.0
Primary ovarian failure	7	28.0
Osteopenia/osteoporosis	14	28.0
Intellectual disability	8	16.0
Cataract	4	8.0

The possible relationship between the occurrence of distant complications and ASD diagnosis was also investigated. An analysis of the relationship between MINI-KID history-based diagnosis and complications rates found that demyelination/hypomyelination and osteopenia/osteoporosis were significantly more common in children with ASD symptoms. Demyelination/hypomyelination occurred in 64.3% of children with MINI-KID-indicated ASD-related symptoms and 36.4% of children without ASD (χ² = 3.85; p = 0.050), whilst osteopenia/osteoporosis occurred in 39.3% of children with ASD and 13.6% of children without ASD (χ² = 4.02; p = 0.045). In both cases, the strength of the correlation as assessed by the φ coefficient was moderate (φ = 0.28) (**Table 8**).

**Table 8:** Frequency analysis with χ^2^ test of independence or Fisher’s exact test for the relationship between MiniKid score and incidence of distant complications of classic galactosemia.

	MiniKid-indicated ASD-related symptoms						
Distant complications	No		Yes				
	*n*	%	*n*	%	χ^2^	*p*	φ
Delayed speech development	9	40.9	15	53.6	0.79	0.374	0.13
Speech disorders	11	50.0	14	50.0	0	1.000	0
Primary ovarian failure	3	20.0	4	44.4	–	0.356	0.26
Intentional/spontaneous tremors	5	23.8	11	39.3	1.31	0.253	0.16
Demyelination/hypomyelination	8	36.4	18	64.3	3.85	0.050	0.28
Osteopenia/osteoporosis	3	13.6	11	39.3	4.02	0.045	0.28
Cataracts	1	4.5	3	10.7	–	0.621	0.11
Intellectual disability	2	9.1	6	21.4	–	0.439	0.17

For the other complications analysed, such as speech disorders, delayed speech development, tremors, ovarian failure, cataracts or intellectual disability, there were no statistically significant differences between the groups of children with and without ASD based on the MINI-KID.

The relationships between the occurrence of long-term consequences of classic galactosemia and the severity of ASD symptoms assessed using the ASRS and ADOS-2 scales were also analysed, but no statistically significant correlations were found.

In the next step, the relationship between distant complications and the diagnosis of ASD made by a children’s psychiatrist on the basis of a comprehensive clinical examination was analysed. In this view, there were no statistically significant differences between the groups with and without ASD in the frequency of any of the complications (**Table 9**).

**Table 9:** Frequency analysis with x^2^ test of independence or Fisher’s exact test for the relationship between ASD diagnosis and incidence of distant complications of classic galactosemia.

	ASD based on psychiatric examination						
Distant complications	No		Yes				
	*N*	%	*n*	%	χ^2^	*p*	φ
Delayed speech development	14	43.8	10	55.6	0.64	0.423	0.11
Speech disorders	17	53.1	8	44.4	0.35	0.556	0.08
Primary ovarian failure	5	25.0	2	50.0	–	0.552	0.20
Intentional/spontaneous tremors	10	32.3	6	33.3	0.01	0.938	0.01
Demyelination/hypomyelination	14	43.8	12	66.7	2.42	0.119	0.22
Osteopenia/osteoporosis	8	25.0	6	33.3	0.40	0.529	0.09
Cataracts	1	3.1	3	16.7	–	0.127	0.24
Intellectual disability	4	12.5	4	22.2	–	0.436	0.13

It was also assessed whether the age of the parents of the study children was related to the presence of an ASD diagnosis. A comparison of mothers’ and fathers’ ages between the groups with and without an ASD diagnosis showed no significant differences (**Table 10**).

**Table 10:** Comparison of parental age depending on ASD diagnosis.

	ASD based on psychiatric examination								
	No (n = 32)			Yes (n = 18)					
Dependent variable	Average rank	*Mdn*	*IQR*	Average rank	*Mdn*	*IQR*	*Z*	*p*	*r*
Mother’s age	26.69	29.00	6.25	23.39	27.50	6.25	-0.77	0.440	0.11
Father’s age	27.98	32.00	9.50	21.08	29.00	7.25	-1.62	0.106	0.23

## 4 Discussion

The present study shows that ASD-related symptoms and clinical ASD diagnoses were frequent in this clinical cohort of children and adolescents with classical galactosemia. The findings are consistent with previous reports indicating that CG is associated with neuropsychological difficulties A clinical diagnosis of ASD was made in 36% of subjects, significantly higher than the frequency observed in the general population (7). In the ADOS-2 protocol, autistic symptoms were found in 48% of children (including 12% in low range, 24% in the moderate range, 12% in the high range), whilst in MINI-KID - in 56% of participants. On the ASRS Scale, 30% of children scored in the elevated, high or very high range, confirming the presence of social-communication difficulties in this group. These data indicate that autistic traits are common in CG, although they do not always meet the criteria for a full clinical diagnosis. The variation between assessment sources is clinically meaningful. The higher rates of ASD-related symptoms identified by ADOS-2 and MINI-KID compared with the final clinical diagnosis may reflect the complex neurodevelopmental phenotype of classical galactosemia. Language impairment, social-communication difficulties, sensory sensitivities and variability in cognitive functioning are common features of CG and may contribute to elevated scores in observational or symptom-based assessment tools. At the same time, the presence of such difficulties does not necessarily indicate autism spectrum disorder. The lower proportion of final clinical ASD diagnoses highlights the importance of integrating developmental history, psychiatric examination, cognitive profile and medical context when evaluating ASD in patients with CG. This multisource approach is particularly important in classical galactosemia, where overlap between ASD-related symptoms and manifestations of the underlying metabolic disorder may complicate diagnostic interpretation (27–29).

The results are consistent with previous studies showing that CG is associated with significant neurodevelopmental deficits including social cognition, emotion regulation and adaptive functioning (13–15, 23). Studies amongst adult patients have documented social difficulties, deficits in emotion recognition and reduced quality of life (16, 17). Available systematic reviews indicate that cognitive impairment in CG is common and persists over time (24), and an IQ meta-analysis confirms that even early treatment does not eliminate the risk of lower intellectual functioning (25). Our findings indicate that social-communication difficulties are common in children and adolescents with classical galactosemia and may resemble features observed in ASD.

In the analysed cohort, lower IQ was associated with greater severity of ASD-related symptoms, particularly in social communication, social-emotional reciprocity and attention. However, ASD-related difficulties were also observed in children with average intellectual functioning. This finding suggests that cognitive variability, which is a well-recognised feature of the classical galactosemia phenotype, may influence the expression and interpretation of ASD-related symptoms, but does not fully explain the social-communication difficulties observed in this population (24, 25). Therefore, elevated ASD-related symptom scores in patients with CG should not be interpreted solely as a consequence of reduced intellectual functioning. Comprehensive clinical assessment remains essential to distinguish ASD from broader neurodevelopmental manifestations associated with classical galactosemia.

Speech and language disorders were a common complication in the study group, confirming previous reports. Patients with CG show an increased risk of articulatory and motor disorders (22) and language delays associated with working memory deficits (26). These symptoms – which include early speech delays, articulatory difficulties, reduced fluency of speech and reduced language flexibility – may resemble features of ASD, especially in social communication and pragmatics, if not assessed in a metabolic context. This reinforces the importance of interdisciplinary diagnosis, integrating neurodevelopmental, metabolic and environmental data.

Analysis of distant complications of CG showed a higher incidence of white matter lesions and osteopenia in children presenting with ASD symptoms, although statistical significance was only obtained in analyses based on MINI-KID. Therefore, these findings should be interpreted as exploratory signals rather than evidence of a direct relationship between these complications and ASD in CG. These observations are part of a growing interest in potential neurobiological mechanisms linking CG complications – such as chronic galactose toxicity, impaired galactose-1-phosphate metabolism or oxidative stress – to myelination processes and the functioning of neuronal networks involved in social processing (5, 6, 30). Neuroimaging studies show numerous structural and functional abnormalities in the brain of CG patients, including white matter lesions and abnormal resting connectivity patterns (31, 32). Although our data do not allow firm conclusions to be drawn, the trends presented suggest potential links that require further research, particularly using uniform ASD assessment protocols and advanced neuroimaging methods.

The study found no significant association between parental age and ASD prevalence, which is in line with population-based analyses indicating that although parental age may be a risk factor for ASD in the general population, its influence is multifactorial and modulated by genetic and environmental factors (33). In CG, metabolic factors appear to play a more important role. However, this finding should be interpreted cautiously because of the limited sample size.

Overall, children and adolescents with classical galactosemia in this clinical cohort frequently presented ASD-related symptoms, and a substantial proportion met criteria for clinical ASD diagnosis. The co-presence of language deficits, cognitive variability and disease-related social-communication difficulties supports the need for systematic psychiatric and psychological assessment in this population. Because the study had no control group, the findings should be interpreted as descriptive clinical frequencies rather than population prevalence estimates.

### 4.1 Study limitations

Despite the use of a broad set of standardised diagnostic tools and the coverage of a large proportion of the paediatric population with classical galactosemia in Poland, the present study has several important limitations that need to be taken into account when interpreting the results.

Firstly, the lack of a control group limits the possibility to directly compare the frequency of ASD symptoms and cognitive functioning profile with the general population. Although this decision was justified on ethical and organisational grounds, it makes it difficult to assess the extent to which the observed difficulties are specific to CG.

Secondly, the cross-sectional nature of the study makes it impossible to analyse changes in symptom severity over time and to determine their relationship to the course of the disease or implemented therapeutic interventions. Longitudinal studies are needed to more accurately determine the trajectories of ASD symptom development in children with CG.

Thirdly, the study did not include own neuroimaging data and the interpretation of potential neurobiological mechanisms is based solely on available results found in literature. This limits the possibility of directly linking the observed symptoms to specific structural or functional changes in the brain of CG patients. Information regarding white matter abnormalities was obtained from previously documented clinical records and was not verified using a standardised neuroimaging protocol conducted specifically for the purposes of the present study.

Fourthly, non-specific language difficulties, speech disorders and sensory hypersensitivity are often present in classical galactosemia, which can elevate the scores of tools used to assess ASD symptoms and lead to the risk of overdiagnosis. These symptoms are characteristic of the CG phenotype and may partly overlap with features observed in ASD. The present study sought to minimise this risk by using multisource diagnostics and interpreting the results in a broad clinical context, but the lack of tools to specifically differentiate these profiles remains a significant limitation.

Fifthly, although the diagnostic tools used vary in terms of sensitivity and specificity, their results were analysed each time as part of a full psychiatric consultation, reducing the risk of misclassification arising from the characteristics of the individual tests. MINI-KID findings were treated as exploratory and were not used as a stand-alone basis for ASD diagnosis.

Finally, although the sample size is relatively large for a rare disease, it remains limited for analyses requiring greater statistical capabilities, particularly with regard to less common complications and their potential association with ASD symptoms.

## 5 Conclusion

1. Symptoms from the autism spectrum are found in children with classical galactosemia much more frequently than in the general population, both in clinical diagnosis and in standardised observational and interview tools. This indicates that autistic traits are an important part of the CG neurodevelopmental phenotype.
2. The differences between the diagnostic tools (ADOS-2, MINI-KID, ASRS) reflect their different specificities and emphasise the need to base the diagnosis of ASD on an integrated clinical assessment, using multiple sources of information.
3. The intellectual functioning of children with CG remains variable, with lower IQ scores correlating with greater severity of autistic symptoms. This indicates the need to consider the cognitive profile when diagnosing ASD in this population.
4. Trends towards a higher prevalence of selected late complications (e.g. white matter lesions) in children with ASD symptoms suggest the need for further investigation of potential neurobiological mechanisms linking CG and atypical socio-communicative development.
5. The results emphasise the importance of systematic, interdisciplinary psychiatric and psychological assessment in children with CG, allowing early identification of social and communication difficulties, and implementation of appropriate forms of therapeutic and educational support.

This study provides new data on the prevalence of autism spectrum symptoms and the neurodevelopmental profile of children with classical galactosemia. The results indicate that CG is associated with an increased risk of social, communication and emotional difficulties, which may occur regardless of the intellectual functioning capacity. This emphasises the importance of early and systematic monitoring of the development of all children with CG in line with current guidelines, with a particular focus on socio-communicative functioning.

In the light of the data presented, it is justified to routinely screen for symptoms of ASD in this population and to refer children early for in-depth psychiatric and psychological diagnostics when worrying signs are present. Comprehensive care for children with CG should involve the collaboration of metabolic, psychiatric, psychological and educational teams, allowing therapeutic and educational interventions to be implemented early on.

Further etiological and longitudinal studies and multi-centre projects using modern neuroimaging methods can significantly expand the understanding of the biological factors underlying socio-communicative difficulties in CG.

## Data Availability

The original contributions presented in the study are included in the article/supplementary material. Further inquiries can be directed to the corresponding author.
